# Adipose tissue explant culture using PDMS flow chambers: an alternative to static explant culture

**DOI:** 10.1080/21623945.2025.2578286

**Published:** 2025-10-24

**Authors:** M Cohen, P Bandaru, K Szylo, N Nguyen, B Nadeak, R Paszkiewicz, JW Ashby, JJ Mack, L Tanaka, J Tan, A Khademhosseini, SD Mittelman

**Affiliations:** a UCLA Children’s Discovery and Innovation Institute, Metabolism Theme, Division of Pediatric Endocrinology, David Geffen School of Medicine; b Terasaki Institute for Biomedical Innovation, Woodland Hills; c UCLA Division of Cardiology, David Geffen School of Medicine

**Keywords:** Adipose, tissue, explant, culture, methods, fluidics, engineering

## Abstract

As obesity rates continue to rise, it is important that we can effectively study adipose tissue to understand its physiological contribution in individuals with obesity. Unfortunately, due to the fragility and buoyancy of adipose tissue, culture remains challenging. *Ex vivo* culture of tissue explants is possible, however after 48 hours explants often display declining viability, increased inflammation, and de-differentiation. Other common approaches include differentiation of preadipocytes and adipocyte isolation by enzymatic dissociation, however these methods are time-consuming and fail to recapitulate the structure and cellular network within adipose tissue. Given these shortcomings, we developed a novel explant culture method using polydimethylsiloxane (PDMS) flow chambers attached to a micro peristaltic pump. This approach reduces air interface while enabling media perfusion, time-resolved measurements of secreted factors, and easy incorporation of treatments. Using our chambers, we assessed viability with resazurin and lactate dehydrogenase (LDH) assays, physiology by measuring glycerol release, architecture by confocal imaging, and retention of adipose gene expression by qPCR. Explants remained viable for over 72 hours. Resazurin reduction was at 84 ± 9% of baseline, and LDH release remained low. Isoproterenol treatment resulted in 2.7 ± 0.5-fold increased glycerol release, while insulin returned release to baseline. Confocal imaging showed preserved architecture, while qPCR of human tissue with insulin and dexamethasone supplementation showed maintained expression of *PPARG* and *FABP4* over 72 hours. Overall, our results suggest PDMS flow chambers are a suitable method for adipose explant culture that requires minimal processing, making this system a viable option for translational research.

## Introduction

White adipose tissue is primarily known as the home for adipocytes – metabolically active cells that store lipids in droplets and release signalling molecules known as adipokines. Additionally, a complex network of other cell types resides in adipose tissue – collectively known as the stromal vascular fraction (SVF) [1]. The SVF includes fibroblasts, endothelial cells, and preadipocytes, as well as a variety of immune cells that play roles in both local adipose tissue and systemic inflammation. Increased inflammation is a major concern in individuals with obesity, contributing to higher incidence of cardiovascular disease, diabetes, and cancer [2].

Considering that obesity is on the rise and shows no signs of ceasing, it is imperative that we have the best tools available to study adipose tissue. The predominant method used to study adipose tissue *in vitro* involves differentiation of primary preadipocytes or immortalized fibroblasts into a monolayer of adipocytes [3]. While this model has proven sufficient for many studies and is both accessible and reproducible, it is important to recognize that cultured adipocytes greatly differ from adipocytes and adipose tissue *in vivo*. Notably, adipocytes *in vitro* contain many lipid droplets (multilocular) while white adipocytes *in vivo* generally only contain one large droplet (unilocular) [3]. Furthermore, *in vitro* models of adipocytes lack SVF cells and the three-dimensional structure of adipose tissue, thus not truly recapitulating adipose tissue *in vivo*. An alternative to differentiated adipocytes is isolation of mature unilocular adipocytes from adipose tissue, however this requires enzymatic digestion and still lacks the SVF component [3]. Thus, if we wish to study adipose tissue as a whole, we must explore other approaches.

Intact adipose tissue explants can be cultured *ex vivo*. Compared to differentiated adipocytes, explants are more physiologically and translationally relevant as they maintain the 3D structure of adipose tissue, the SVF network, and patient characteristics. They also require less processing and do not require lengthy differentiation. However, explants rapidly de-differentiate, lose viability, and become inflamed [4]. Additionally, explant culture presents numerous technical challenges, such as buoyancy, air-interface, fragility, risk of contamination, and difficulty when changing media or collecting supernatant for analysis. Thus, we sought to develop an alternative method for explant culture that addresses these shortcomings using polydimethylsiloxane (PDMS) flow chambers.

## Results

### Chamber design

We elected to make chambers from PDMS, a mouldable gas-permeable polymer frequently used in microfluidic culture chambers for many cell and tissue types [5,6]. To maintain the 3D structure and SVF component of adipose tissue, explants in chambers were minced until tissue pieces were approximately 1 mm^3^ in size.

**Figure 1.**
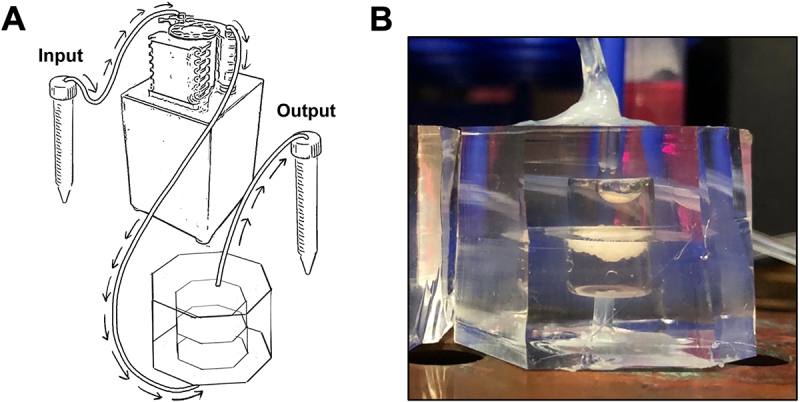
Novel flow chamber design. A) Schematic diagram of flow chamber assembly in incubator. Arrows indicate direction of flow. B) Photograph of assembled chamber in incubator containing mouse adipose tissue.

To address the issue of buoyancy, the centre of the flow chamber housed a polycarbonate membrane with 0.4-micron pores (Figure 1). This pore size allowed for media and secreted factors to exit the chamber but not cells. Additionally, it reduced air interface of the adipose tissue since the chambers above and below the tissue remained filled with media throughout the duration of the experiment.

Another concern of static culture is the need for frequent media changes, which can be tricky due to the delicate nature of adipose tissue. To address this, flow chambers were connected to a mini peristaltic pump, which constantly drew media from a reservoir tube and replenished media for the tissue. This allowed for media changes without disturbing the tissue, as well as clearance of depleted media that accumulates in the chamber. While flow rate is adjustable, we used the lowest flow rate setting allowed on the ESI Pump Network software (114 µl/hour) so adipose tissue is subjected to minimal shear stress. This flow is similar to, albeit slightly lower than human capillary flow rate in adipose tissue *in vivo*, which has been shown to range from 3–6 ml/minute/100 g tissue depending on prandial state [7].

In addition to our chamber allowing for better culture conditions for adipose tissue explants, there also are experimental advantages to our system. Since input media can be changed without needing to make direct contact with the tissue, this allows for seamless integration of drug treatment and reduced contamination risk. Our system also allows for easy generation of adipose tissue conditioned media for use with other cell types in culture. Furthermore, since our system outputs effluent media at a consistent rate, this allows for time-resolved measurements of secreted factors for time course experiments. The only caveat is that one must consider the delay from when input media is changed to when it reaches the chamber and output tube. This delay can be estimated from flow rate and volume, or measured directly, for example by using a colorimetric dye. Based on the flow rate of 114 µl/hour, combined tube length, tube diameter, and chamber volume of 500 µl (minus ~100 µl of tissue), we estimated an ~12-hour delay before media change was reflected in effluent. However, shorter tubing can be used if needed depending on incubator configuration. Additionally, it is possible to briefly increase the flow rate to accelerate the passage of input media into the chamber, thus reducing delivery delay.

### Explant viability

**Figure 2.**
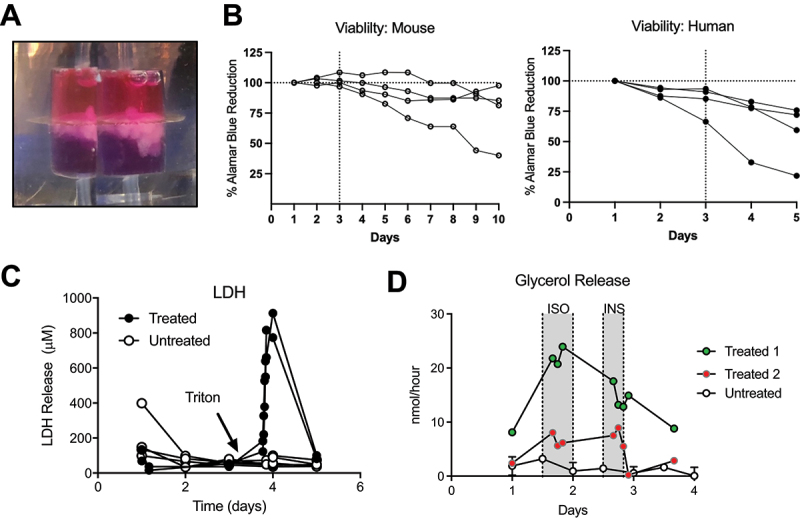
Adipose tissue retains viability and function in flow chambers. (A) Photograph of chamber containing mouse tissue and alamarBlue viability dye. Blue media below adipose tissue is oxidized form, while pink media above adipose tissue has been reduced by the viable tissue (B) Viability represented by percent alamarBlue reduction by human and mouse adipose tissue. Dashed line at 3 days represents typical culture duration. Mouse *N* = 4, human *N* = 4. (C) LDH release (µM) over 120 hours from mouse epididymal adipose tissue explants. Arrow denotes when 0.2% TX-100 detergent was added to chamber to accelerate cell death. Untreated *N* = 5, treated *N* = 2. (D) Glycerol release in human explants (nmol/hour) over 96 hours. Shaded region represents when samples were exposed to 10 µM isoproterenol (ISO) and 100 nM insulin. Untreated *N* = 4, treated *N* = 2. Error bars = standard deviation.

To determine how long adipose tissue would maintain viability in our flow chambers, we infused alamarBlue HS (high sensitivity) resazurin viability dye in the media and quantified reduced dye in the effluent (Figure 2(A,B)). Freshly harvested epididymal white adipose tissue explants from 17–20-week-old C57/BL6J 60% diet induced obesity (DIO) male mice or remnant human peri-colonic white adipose tissue explants were perfused with RPMI 1640 complete media containing 10% alamarBlue HS substrate in chambers and monitored. All mouse adipose tissue and three of the four human samples remained highly viable ( > 85%) for 72 hours, with minimal loss of reduction capacity during that time frame (Figure 2(B)). In fact, three of four mouse adipose tissue samples retained reduction capacity > 85% for 6 days in culture. The longer period of viability of mouse tissue may be due to more rapid processing prior to putting in the chamber, or inherent differences between species.

Next, we quantified cell death by lactate dehydrogenase (LDH) release in media effluent. LDH release (µM) from mouse adipose tissue remained at a constant low level, implying that while some cell death was evident as expected, rate of release was stable in ambient culture conditions (Figure 2(C)). When cell death was induced using 0.2% TX-100 detergent in media after 96 hours, LDH release spiked to values > 10X ambient levels, confirming that an abundance of live cells was still present.

### Adipose tissue function

Adipose tissue cultured *ex vivo* can rapidly de-differentiate and lose function [4]. Thus, we next tested whether human adipose tissue explants exhibited lipolysis, and whether this was responsive to known *in vivo* modulators, as lipolytic activity is a hallmark of adipose tissue function [8,9].

In peri-colonic adipose tissue explants, glycerol release (nmol/hour) remained stable in ambient conditions for 96 hours in RPMI 1640 complete media, suggesting a relatively constant rate of lipolysis over time (Figure 2(D)). Addition of the beta agonist isoproterenol (10 μM) resulted in increased glycerol release compared to ambient conditions, while exposure to porcine insulin (100 nM) suppressed glycerol release after isoproterenol treatment. One peri-renal adipose explant showed low baseline glycerol release and no response to isoproterenol or insulin (not shown). Whether this was due to the different adipose tissue origin or other experimental variables is unclear. Overall, our data suggest that baseline lipolysis of peri-colonic human explants in chambers remains relatively low in ambient conditions, and explants are functionally responsive to induction/inhibition by classical activators.

### Confocal imaging

**Figure 3.**
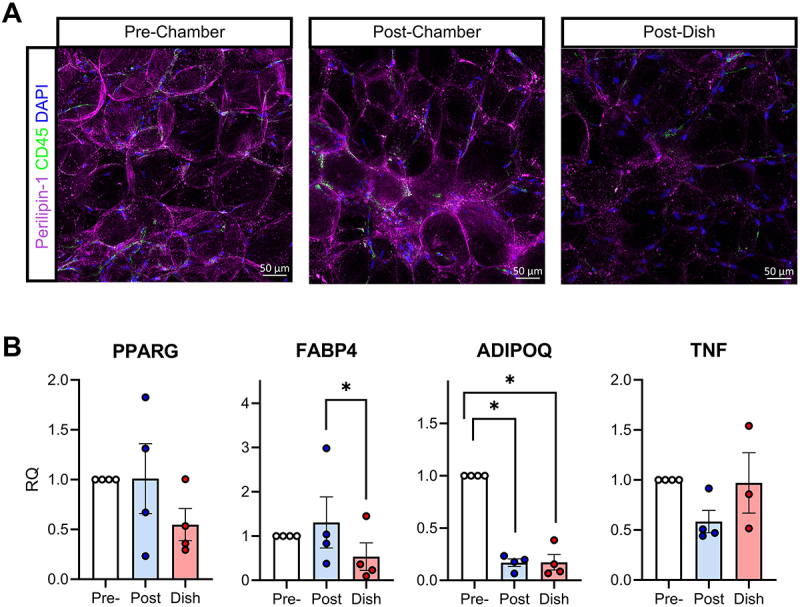
(A) Representative confocal images of whole mount PFA- fixed human adipose tissue at time of tissue collection (0 hours, pre-chamber) after 120 hours an in flow chamber, and after 120 hours in static dish culture. Purple = PLIN1 (lipid droplet), green = CD45 (immune cells), blue = DAPI (nucleus). (B) RT-qPCR of adipose tissue hallmark genes in human peri-colonic adipose tissue explants before incubation (pre), 72 hours in flow chambers (post), and 72 hours in static culture (dish). Explants were cultured in M199 containing 7 nM insulin and 25 nM dexamethasone. Target gene expression was normalized to *POLR2A* housekeeping gene expression. *N* = 4 except dish TNF *N* = 3. Error bars = standard deviation. Paired two-tailed t-test was used with Tukey’s adjustment for multiple comparisons: * = p ≤ 0.05.

In addition to viability and functionality, we sought to determine whether adipose tissue in flow chambers retained its 3D structure better than explants in static culture. Human adipose tissue explants were processed as described above and split into ~100 mg aliquots. One aliquot was cultured in a flow chamber while the other was placed in static culture in a 24 well plate containing 500 µl of media. Both conditions used RPMI 1640 complete media for 3, 5, and 9 days (*N* = 1 for each time point). Regions of interest that demonstrated uniform staining considered representative of the whole specimen were located and visualized using confocal microscopy (Figures 3(A), S2). Perilipin 1 (PLIN1) staining confirmed the maintenance of many adipocyte unilocular lipid droplets in adipose tissue in flow chambers in all paired samples [10]. By contrast, lipid droplet architecture was more noticeably lost in adipose explants in static culture. It is important to note that due to limited sample size, PLIN1 staining was used as a qualitative measure and was not quantified based on intensity. CD45 staining confirmed retention of immune cells in explants, however signal was variable among samples and was more prominent when viewed at higher magnification (Figures 3(A), S3). Thus, our results suggest flow chambers better preserve adipose tissue structure compared to traditional static culture methods, even for extended duration in general-use media.

### Gene expression with supplements and media 199

Our final question was whether adipose tissue gene expression was retained during culture in flow chambers. We selected a panel of hallmark adipocyte genes to analyse: Peroxisome Proliferator Activated Receptor Gamma (*PPARG*), Adiponectin (*ADIPOQ*), and Fatty Acid-Binding Protein 4 (*FABP4*). Tumor Necrosis Factor alpha (*TNF*) was also included as a measure of explant inflammation. Expression of adipocyte markers in both mouse and human adipose tissue in RPMI 1640 complete media decreased significantly over the 72 hours in the flow chamber (Figure S1). This decrease was similar between flow chamber and dish samples, indicating that while our chamber system maintained viability and tissue architecture in general use media, it did not prevent dedifferentiation or *TNF* induction.

To test whether dedifferentiation and inflammation could be reduced in media optimized for adipose tissue explants, we performed additional experiments in serum-free Media 199 (M199) supplemented with 7 nM human insulin and 25 nM dexamethasone. This media formulation has been shown by Fried et al. to preserve adipose tissue explants *ex vivo* [11]. Additionally, we selected *POLR2A* as a new housekeeping gene for normalization since it has been shown to be stable in human visceral adipose tissue and demonstrated more consistency than beta actin (*ACTB)* which we had been using [12]. Overall, M199 + insulin and dexamethasone resulted in improved adipose tissue gene expression compared to RPMI (Figure 3(B)). *PPARG* and *FABP4* expression was well-maintained by explants in flow chambers compared to those in traditional dish culture, though *ADIPOQ* expression showed a similar decline in chamber and dish. *TNF* expression in flow chambers was lower than pre-chamber, though this was likely due in part to the presence of dexamethasone in the media, as glucocorticoids have been shown to suppress TNFα production [13]. Overall, our results suggest that explants in flow chambers using M199 with insulin and dexamethasone better maintain gene expression of classic adipocyte genes than those in static culture.

## Discussion

In the present study, we introduce a novel culture method of adipose tissue explants using PDMS flow chambers. While perfusion of isolated adipocytes was first reported in rat adipocytes by Turpin et al. in 1977 [14], to our knowledge our system is the first to incorporate intact, minimally processed human adipose tissue explants that retain tissue architecture. Our system addresses notable pitfalls of static explant culture such as buoyancy of tissue resulting in a tissue-air interface, high metabolic demand of tissue resulting in spent media, and technical challenges with culture due to tissue fragility. Additionally, our system allows for simple measurement of secreted factors from tissue, media conditioning, and seamless introduction of treatments without direct contact with the tissue. We demonstrated that mouse and human adipose tissue in flow chambers maintained viability, three-dimensional architecture, and physiological control of lipolysis over at least 72 hours. While adipose tissue showed evidence of adipocyte dedifferentiation in RPMI complete media, this was greatly reduced in M199 plus dexamethasone and insulin. Although adiponectin expression still decreased in M199 media, this may have been due to dexamethasone, which has been shown to suppress adiponectin expression in both explants and cultured adipocytes [15]. Overall, our system provides an attractive alternative to static adipose tissue explant culture when studying intact adipose tissue *ex vivo*.

One consideration regarding experiments conducted on our chamber was the choice of media, as we elected to use RPMI 1640 with 10% FBS for tissue viability validation experiments. While we recognize that there are more suitable serum-free media formulations for adipose tissue-only experiments, such as M199 with dexamethasone and insulin, we tested RPMI 1640 as this media would allow for culture of other cell types either in the flow chambers with adipose tissue or in conditioned media effluent from flow chambers. Additionally, the inclusion of dexamethasone and insulin, while optimizing adipose homoeostasis, could have off-target effects in co-cultured or conditioned media-treated cells and confound results. Investigators using this system will have to choose their media composition based on their specific needs.

Another consideration is the fact that PDMS has been shown to absorb small molecules in some applications, which may have consequences in experimental outcomes [16]. Our results suggest that all compounds used in our study were adequately delivered to the cells, as their intended effects were observed, however we did not conduct any quantitative measurements to determine any absorption of compounds that may have occurred. Thus, precaution must be taken when designing experiments using small molecules in our flow chambers.

We also recognize that the data presented is limited in sample quantity. The goal of the present study is to demonstrate the functionality of our design and ability to maintain live tissue for an extended duration. Understandably more data are needed to validate use for specialized applications and assays. Experiment-specific parameters for future optimization include determination of ideal tissue size and optimal flow rate. Due to the simple design of our chambers, ultimately the final product would be scalable in size, modular, re-usable, and easy to assemble – making the system accessible and potentially useful with other primary tissue types in addition to adipose. Our model today, while preliminary in nature, serves as a blueprint for future developments.

Our system adds a new alternative to the numerous other approaches developed in recent years that steer away from multilocular differentiated adipocyte models and provide more translational alternatives. A microfluidic adipocyte-on-a-chip system has been developed that allows for culture of small populations of mature unilocular adipocytes obtained by collagenase digestion [17]. Cells in this system remain viable and functional for an extended period, and due to chamber design cells can be imaged in-chamber by microscopy. This system provides a physiologically relevant platform for functional and pharmacological testing in unilocular adipocytes, however lacks the SVF component our flow chamber provides. Adipose spheroids derived from differentiated cells have also become an appealing approach, allowing for more physiologically relevant 3D recapitulation of adipose tissue *in vitro* [18]. Newer spheroid models have even been developed to have a vascular component, making these systems more representative of adipose tissue *in vivo* [19]. Another novel method developed known as membrane mature adipocyte aggregate cultures (MAAC) uses porous trans-well membranes and cultures primary isolated mature adipocytes beneath [20]. These cells are highly viable, functional, and can even be trans-differentiated to brown-like adipocytes. This approach requires enzymatic digestion and lacks SVF but provides an excellent option for studying unilocular adipocytes *in vitro*. Our method, as well as others mentioned above, represent the overarching goal of refining the study of adipose tissue, thus providing better insight into tissue function in both healthy and diseased states.

## Methods

### Chamber fabrication

The mould for the PDMS flow chamber was cut out from ¼ inch acrylic sheets using a CO_2_ laser and then transferred to a round Petri dish to form. SYLGARD 184 elastomer (Dow Chemicals) was mixed with the crosslinker at a 10:1 ratio and mixed thoroughly, then degassed in a desiccator using a vacuum pump. The mould was transferred to an 80°C oven for curing for at least 2 hrs. The PDMS disks were then manually removed from the mould and the inlet and outlets made using a biopsy punch. Clear PET membranes with 0.4 µm pores were cut out from cell culture inserts (Falcon 353,090) and placed between the two 500 µl PDMS wells, then bonded using O_2_ plasma for 100 seconds and cured overnight at 80°C. Chambers were then cut to the desired shape and autoclaved to ensure sterility (Figure 1).

### Chamber assembly

26 cm long 0.76 mm×2.286 mm OD Tygon tubing (Cole-Parmer) was attached to 30 cm long 0.76 mm flared mini peristaltic pump connectors (Elemental Scientific) and sealed with 5-minute epoxy (Devcon). Adipose tissue samples were added to the chamber through the hole on the bottom with a cut 1 mL pipette tip. Remaining volume in the bottom and top chambers was filled with media using a syringe. Once the sample was in the chamber, tubing with the peristaltic connector was attached to the input side of the chamber, and an additional piece of tubing (26 cm in length) was added to the output side of the chamber. Both sides were sealed with epoxy to prevent leakage. Chambers were then transferred to an incubator set at 5% CO_2_ and 37°C, attached to the peristaltic pump (Elemental Scientific), and placed upright. Using a 27 g needle and syringe, media was manually infused into the input line to clear air bubbles, then input tubing was placed in media in a 15 ml conical tube with a hole in the lid. Output tubing was placed in the desired tube to collect effluent. Once assembly was complete, the peristaltic pump was run at 114 µl/hour flowrate using ESI Pump Network software (Elemental Scientific). RPMI 1640 complete media containing 10% FBS (Omega Scientific), 1% GlutaMax (Gibco), 1% sodium pyruvate (Gibco), 0.1% Gentamicin (Gibco) was used for all experiments except optimization of gene expression, which used Media 199 with Earle’s salts (Gibco) plus 7 nM human insulin (Sigma), 25 nM dexamethasone (Sigma), and 0.1% Gentamicin (Gibco).

### Human sample collection

Human peri-colonic adipose tissue remnant samples were collected by the UCLA Translational Pathology Core Laboratory after obtaining written informed consent (IRB-11–2504). Distribution of samples to our laboratory was anonymized and included no protected health information, and therefore was not considered human research. Samples were placed in in RPMI 1640 media with no additives upon arrival at the TPCL and maintained at 4°C until processing.

### Mouse sample collection

Diet-induced obese male 17–20-week-old C57BL/6J mice raised on a 60% calorie from fat diet were obtained from Jackson Laboratories. Mice were sacrificed using CO_2_ euthanasia. Immediately following euthanasia, epididymal white adipose tissue was collected and placed in PBS on ice. Animal studies were conducted in adherence to ARRIVE guidelines.

### Adipose tissue preparation

To prepare tissue for addition to the flow chamber, 100 mg of tissue was weighed and transferred to PBS. To limit inclusion of red blood cells, explant pieces were chosen that contained little to no major vasculature. Tissue was then minced with iris scissors until pieces were approximately 1 mm^3^ in size. Following mincing, tissue was washed 3X with media in a 1.5 ml Eppendorf tube and then transferred to the flow chamber or a well in a 48-well culture plate with 500 μl of media, which is the approximate volume of the flow chamber compartment. No red blood cells were evident upon transfer.

### AlamarBlue resazurin assay

AlamarBlue HS reagent (Invitrogen, Thermo Scientific) was diluted 1:10 in RPMI 1640 media with 10% FBS, 1% GlutaMax (Gibco), and 1% sodium pyruvate (Gibco) to use as input media. Chambers were run as described above. Input and effluent were collected every 24 hours and absorbance was quantified at 570 nm and 600 nm using a NanoDrop One spectrophotometer (Thermo Scientific). Values were then input into the following equation to calculate percent reduction:

$$\frac{\left(117216\;x\;\mathrm{Effluentabs}.570nm\right)-\left(80586\;x\;\mathrm{Effluentabs}.600nm\right)}{\left(155677\;x\;\mathrm{Inputabs}.600nm\right)-\left(14652\;x\;\mathrm{Inputabs}.570nm\right)}\times \;100$$

### Lactate dehydrogenase (LDH) assay

Effluent samples from the flow chamber were snap-frozen in liquid nitrogen and stored at −80°C until analysis using the LDH-glo kit (Promega) according to the manufacturer’s protocol. Samples were quantified using the Clariostar plate reader (BMG Labtech). In some experiments, TX-100 detergent was added to the flow chamber input media at a concentration of 0.2% to induce cell death.

### Glycerol release assay

Flow chamber effluent samples were analysed using colorimetric glycerol assay (Millipore Sigma) according to manufacturer protocol. Absorbance was quantified using the Clariostar plate reader (BMG Labtech) and values were obtained based on a standard curve generated according to manufacturer protocol. In some experiments, 10 μM isoproterenol (Sigma-Aldrich) and 100 nM porcine insulin (Sigma-Aldrich) were serially added to the input media.

### Collection of tissue from flow chamber

Upon completion of experiments, flow chambers were removed from the peristaltic pump. Working quickly to avoid air exposure, chambers were cut open using a razor blade and adipose tissue was collected using a cut pipette tip or forceps. Tissue was then transferred either to PBS for imaging or snap frozen in LN_2_ for RNA isolation.

### Confocal imaging of whole mount adipose tissue

Adipose tissue from before or after chamber/dish culture were fixed in 4% PFA in 1X PBS overnight. Samples were then washed and stained with rabbit anti-Perilipin 1° antibody (Abcam ab61682) and mouse anti-Cd45 1° antibody (Cell Signaling 13917T), followed by Alexa Fluor 488 (Abcam ab150105) and Alexa Fluor 647 (Abcam ab150135) secondary antibodies and DAPI nuclear stain (AAT Bioquest #17507). Once stained, samples were whole mounted on glass slides with ProLong Gold mounting media (Invitrogen P36930). Images were acquired using a ZEISS LSM900 confocal microscope with 20x Plan-Apochromat objective. Identical laser settings were applied to samples being compared, however samples were not blinded. After acquisition, a maximum intensity projection of the Z- stack was applied using ZEN Blue 3.5 software (ZEISS).

### RT-qPCR

RNA from adipose tissue samples was isolated using the RNEasy Lipid Kit (Qiagen) according to the manufacturer’s protocol. In brief, samples were added to Qiazol lysis reagent (Qiagen) and homogenized using zirconium beads and the BeadBlaster system (Benchmark Scientific). Once homogenized, RNA was separated using chloroform and isolated using RNEasy spin columns (Qiagen). Purified RNA was quantified using a NanoDrop One spectrophotometer (Thermo Scientific)

cDNA was prepared using the High Capacity cDNA Reverse Transcription Kit (Applied Biosystems). RT-qPCR reactions were prepared using PowerUp SYBR green master mix (Applied Biosystems) and data were acquired using the QuantStudio 6 Flex system (Applied Biosystems) using the 2^–∆∆Ct^ method. Primer sequences (Integrated DNA Technologies) are listed in Tables S1 (human) and S2 (mouse) [21]. Statistical analyses consisted of two-way ANOVA followed by paired two-tailed t-tests with Tukey’s correction for multiple comparisons conducted on GraphPad Prism.

## Data Availability

The data that support the findings of this study are openly available in Dryad at https://doi.org/10.5061/dryad.866t1g22t. (This link will become active following the peer review process. For peer review purposes the data can be accessed using the following link: http://datadryad.org/share/wg64K4CJzR0YvDOShwXW6DV3GCsQSdlh4rnF2GeoF2Q)
